# The potential value of fibrinogen to albumin ratio (FAR) in the assessment of inflammation in spondyloarthritis

**DOI:** 10.1186/s12891-022-05797-6

**Published:** 2022-09-15

**Authors:** Yimei Ding, Luan Xue

**Affiliations:** Department of Rheumatology and Immunology, Yueyang Hospital of Integrated Traditional Chinese and Western Medicine, Shanghai University of Traditional Chinese Medicine, Shanghai, 200437 China

**Keywords:** Spondyloarthritis, Psoriatic arthritis, Reactive arthritis, Ankylosing spondylitis, Fibrinogen to albumin ratio

## Abstract

**Background:**

Fibrinogen to albumin ratio (FAR) is a newly investigated indicator for inflammation. The study aimed to explore the potential ability of FAR in assessing the severity of inflammation in spondyloarthritis.

**Methods:**

The clinical data of 196 spondyloarthritis (SpA) patients, 66 osteoarthritis (OA) patients, and 81 healthy controls (HC) were collected in this retrospective study. The SpA group included 69 psoriatic arthritis patients, 47 reactive arthritis patients and 80 ankylosing spondylitis patients. Chi-square test and Mann–Whitney U test, Spearman’s correlation test, regression analysis, and ROC analyses were used for the analysis of FAR.

**Results:**

FAR level in group SpA was higher than in OA or HC. In the SpA group, the reactive arthritis group was characterized by the highest FAR level. After matching the erythrocyte sedimentation rate, a significant difference occurred between groups SpA and OA, but not in SpA subgroups. The FAR level was significantly related to erythrocyte sedimentation rate and C-reactive protein. After regression and receiver operating characteristics analysis, FAR was considered the most potential pointer to evaluate inflammation in SpA with the area under curve of 0.95. The recommended cut-off value of FAR was 9.44 for serious inflammation and 8.34 for mild conditions.

**Conclusion:**

FAR is closely related to inflammatory biomarkers and can be a potential indicator in the assessment of inflammation in spondyloarthritis.

**Supplementary Information:**

The online version contains supplementary material available at 10.1186/s12891-022-05797-6.

## Introduction

Spondyloarthritis (SpA) is composed of a group of inflammatory diseases affecting the joints and spine, such as psoriatic arthritis (PsA), arthritis related to inflammatory bowel disease (IBDA), reactive arthritis (ReA), a subgroup of juvenile idiopathic arthritis (JIA), and ankylosing spondylitis (AS) [1]. The prevalence of SpA ranged from 14 to 43 per 10,000 persons [2]. Spondyloarthritis presents as a long-term and recurrent disease. AS, the prototypic and best-studied subtype, is frequently associated with significant pain and disability. Non-steroidal anti-inflammatory drugs (NSAIDs) and biologics such as TNF blockers are commonly used to alleviate pain and avoid disability [3]. Nevertheless, 2016 ASAS-EULAR management recommendations suggest that continuous NSAIDs treatment should be based on symptomatic response [4]. Thus, the assessment of disease activity is of vital importance. Different scales, including Ankylosing Spondylitis Disease Activity Score (ASDAS), Bath Ankylosing Spondylitis Disease Activity Index (BASDAI), and Disease Activity Index for Psoriatic Arthritis (DAPSA), are developed to estimate it [5, 6]. ASDAS, the most commonly used scale, is composed of subjective symptoms expressed by the Visual Analogue Scale (VAS) and objective inflammatory biomarkers. The serum levels of C-reactive protein (CRP) and erythrocyte sedimentation rate (ESR), reflecting inflammation, have been confirmed to be associated with disease activity and are now generally used in the assessment [7]. Except for the two traditional markers, new indicators are in exploration. Albumin (ALB) is not only a referent of nutritional status but also shows its value in predicting the seriousness of inflammation [8]. As reported, fibrinogen (FIB) may be a reliable pointer to disease activity in AS [9]. Furthermore, the fibrinogen to albumin ratio (FAR) presents more specificity and sensitivity in evaluating disease activity [10]. In addition to AS, FAR could be valuable markers of ongoing inflammation and joint affection detected by musculoskeletal ultrasonography in rheumatoid arthritis [11].

Nonetheless, the discriminability of FAR in assessing the severity of inflammation between various arthritis or different SpA subgroups remains obscure. In this study, a comparison between group SpA and OA aims at the first question, while an exploration between group AS, PsA and ReA aims at the second. Analyses after CRP or ESR matching aim to investigate the discernibility ability of FAR when the level of inflammation is similar. Then the study investigates the correlation between FAR and CRP/ESR, a subjective part of the criteria in evaluating disease activity, to search for the potential value of FAR in the assessment of spondyloarthritis. In addition, the ability of possible indicators is meticulously examined to select the optimal one and figure out the recommended cut-off value.

## Materials and methods

### Patients and clinical data

This study was designed as a single-center, retrospective study with 343 cases involved. All patients’ data were from Yueyang Hospital of Integrated Traditional Chinese and Western Medicine, Shanghai University of Traditional Chinese Medicine, with a complete medical history including symptoms and blood examination from February 2014 to September 2021. These patients were clinically diagnosed with ankylosing spondylitis, psoriatic arthritis, reactive arthritis, and osteoarthritis by a professional rheumatologist and respectively fulfilled the 2009 update of spondyloarthritis international society classification criteria for axial spondyloarthritis [12], classification of psoriatic arthritis criteria [13], criteria for reactive arthritis [14], and EULAR recommendations for the diagnosis of knee osteoarthritis [15].

Any of the following situations were excluded to control bias. (1) Patients with other rheumatic diseases. (2) Patients with severe nephropathy, hepatopathy, and hemopathy, which may relate to the generation and excretion of albumin and fibrinogen. (3) Patients with acute infection.

The selection of patients was based on the following rules. (1) All PsA, ReA, and OA inpatients were included without selection. (2) AS patients were randomly selected from all AS inpatients. Gender-age matching between PsA, ReA and AS was not adopted because it could increase the risk of bias. For example, if AS patients were matched to ReA, the average course of these matched AS patients would be longer than that of all AS patients. Increased average course could lead to severe disease activity and introduce additional bias.(3) Healthy controls were from health examination data and were matched to SpA patients in age, gender and white blood cell (WBC) counts.

### Clinical and laboratory evaluation

The basic data was composed of basic information (age and gender), infection indicators (white blood cell counts (× 10^9/L) and lymphocytes counts(× 10^9/L)), inflammation indicators (CRP (mg/L) and ESR (mm/L)), HLA-B27 status, and the focused indicators (albumin(g/L), fibrinogen(g/L), and FAR). ESR and HLA-B27 were absent in healthy controls because the tests were not necessary in a basic medical examination.

In the study, a hyper-inflammatory state was defined as CRP > 33.88 mg/L or ESR > 52 mm/h. The standard was calculated by ASDAS-CRP and ASDAS-ESR when a subjective assessment was valued as 4 and a total score > 3.5, which suggested high disease activity [16]. A slight inflammation was defined as whether CRP > 10 mg/L or ESR > 20 mm/L, which was above the normal range under the standards of this research institution.

### Statistical analyses

Quantitative variables were reported as mean ± standard deviation (SD), and categorical variables were described as ratios and percentages. Basic data were analyzed by Mann–Whitney U test for abnormally quantitative data (age, WBC, lymphocyte, ESR, CRP, albumin, fibrinogen, and FAR) and chi-square test for categorical data (gender and HLA-B27 status). Correlation analyses between albumin, fibrinogen, FAR and ESR, CRP were evaluated by Spearman’s correlation test. Univariate linear regression was used to screen for possible predictors from a series of indicators. The series of indicators were chosen by a rheumatologist according to clinical practice. Factors with *P*-value < 0.0001 in univariate regression were selected as possible predictors, which included WBC, ALB, FIB and FAR. Then multi-factor linear regression and stepwise regression were reported to confirm the significance of possible indicators. Because FAR is the ratio of FIB to ALB, the multivariate regression and stepwise regression were separately applied to FAR and to FIB, ALB. Receiver operating characteristics (ROC) analysis was used to estimate the ability of FAR in reflecting hyper-inflammatory state and identifying cut-off values of FAR in overall consideration of sensitivity and specificity. The choice of cut-off value was based on Youden Index. In the study, FAR was calculated by FAR = fibrinogen/albumin× 100 to keep more effective digits. Multiple imputation and regression imputation were adopted to fill in the missing data to keep as many samples as possible while avoiding miscalculation. *P*-value < 0.05 was considered significant. Groups were matched by SPSS. Multigroup analysis was corrected by SNK test before being reported.

## Results

### Clinical and laboratory characteristics

Finally, 196 patients with SpA, 66 patients with OA, and 81 healthy controls were collected in this retrospective study. The SpA group included 69 patients with PsA, 47 patients with ReA, and 80 patients with AS. The highest percentage of missing data was in lymphocyte counts, which was 7.8%. The missing percentages of CRP, ESR, ALB, FIB, and FAR were less than 5%. The basic characteristics of the SpA group, OA group, and HC group are shown in Table 1, while the information of group PsA, ReA and AS are shown in Table 2. An apparent difference between the SpA group and OA group indicated the distinction in pathology and epidemiology. SpA patients performed higher CRP, ESR, FIB levels, and lower ALB levels in serum (*p* < 0.05), which supported that SpA patients undergone more serious inflammatory reactions. Though the HC group was matched to the SpA group by gender, age and WBC counts, still revealed divergence in CRP, ESR, FIB, ALB, and FAR (*p* < 0.05). Within SpA patients, three subgroups (group PsA, ReA, and AS) showed discrepancies in basic information and inflammatory biomarkers. The ReA group was characterized by advanced age, a high incidence in women, and serious inflammatory reactions. AS, just as reverse, occurred more frequently in young men with lower CRP, ESR and FAR levels (*p* < 0.05). The PsA group, with the lowest mean value in ESR, FIB, and FAR, had no significant difference from the AS group.

**Table 1:** Clinical characteristics of SpA patients, OA patients and healthy controls

Characteristics	SpA (*n* = 196)	OA (*n* = 66)	HC (*n* = 81)	Reference	*P* value	
					P_1_	P_2_
Age (years)	50.73 ± 1.28	64.07 ± 1.73	50.94 ± 1.68	/	< 0.001	0.913
Gender (Male/Female)	120/76	14/52	49/32	/	< 0.001	0.910
CRP (mg/L)	27.57 ± 2.84	4.00 ± 1.04	2.78 ± 0.31	0–10	< 0.001	< 0.001
ESR (mm/L)	47.47 ± 2.77	19.66 ± 1.98	/	0–20	< 0.001	/
WBC(× 10^9^/L)	7.18 ± 0.17	5.82 ± 0.20	7.47 ± 0.24	3.5–9.5	< 0.001	0.250
Lymphocytes(×10^9^/L)	1.87 ± 0.06	1.86 ± 0.09	1.86 ± 0.07	1.1–3.2	0.807	0.797
Albumin(g/L)	38.64 ± 0.36	40.75 ± 0.54	43.42 ± 0.40	35–50	< 0.001	< 0.001
Fibrinogen(g/L)	3.84 ± 0.10	2.68 ± 0.89	2.66 ± 0.55	2–4	< 0.001	< 0.001
FAR	10.22 ± 0.29	6.58 ± 0.24	6.17 ± 0.14	/	< 0.001	< 0.001
HLA-B27 (positive/negative)	86/102	5/47	/	/	< 0.001	/

(P_1_: difference between SpA and OA; P_2_: difference between SpA and HC

*SpA* spondyloarthritis, *OA* osteoarthritis, *HC* healthy controls, *WBC* white blood cells, *FAR* fibrinogen to albumin ratio, *CRP* C-reactive protein, *ESR* erythrocyte sedimentation rate)

**Table 2:** Clinical characteristics of PsA patients, ReA patients and AS patients

Characteristics	PsA (*n* = 69)	ReA (*n* = 47)	AS (*n* = 80)	Reference	*P* value		
					P_1_	P_2_	P_3_
Age (years)	52.94 ± 1.72	58.07 ± 2.58	43.85 ± 1.72	/	0.313	0.002	0
Gender (Male/Female)	41/28	18/29	61/19	/	0.037	0.034	0
CRP (mg/L)	23.47 ± 3.84	49.14 ± 6.36	21.82 ± 3.20	0–10	0	1	0
ESR (mm/L)	41.12 ± 4.19	55.72 ± 4.55	45.80 ± 3.68	0–20	0.013	0.737	0.168
WBC(×10^9^/L)	7.07 ± 0.24	7.83 ± 0.53	7.02 ± 0.20	3.5–9.5	1	1	1
Lymphocytes(×10^9^/L)	1.78 ± 0.08	1.73 ± 0.15	1.97 ± 0.08	1.1–3.2	1	0.509	0.180
Albumin(g/L)	38.75 ± 0.62	35.32 ± 0.59	39.99 ± 0.45	35–50	0.001	0.54	0
Fibrinogen(g/L)	3.70 ± 0.16	4.40 ± 0.22	3.71 ± 0.14	2–4	0.008	1	0.015
FAR	9.33 ± 0.56	10.52 ± 0.87	9.50 ± 0.38	/	0.001	1	0.001
HLA-B27 (positive/negative)	11/54	4/41	71/7	/	0.27	0	0

(P_1_: difference between group PsA and ReA; P_2_: diferrence between group PsA and AS; P_3_: diferrence between group ReA and AS

*PsA* psoriatic arthritis, *ReA* reactive arthritis, *AS* ankylosing spondylitis, *WBC* white blood cells, *CRP* C-reactive protein, *ESR* erythrocyte sedimentation rate, *FAR* fibrinogen to albumin ratio)

**Fig. 1:**
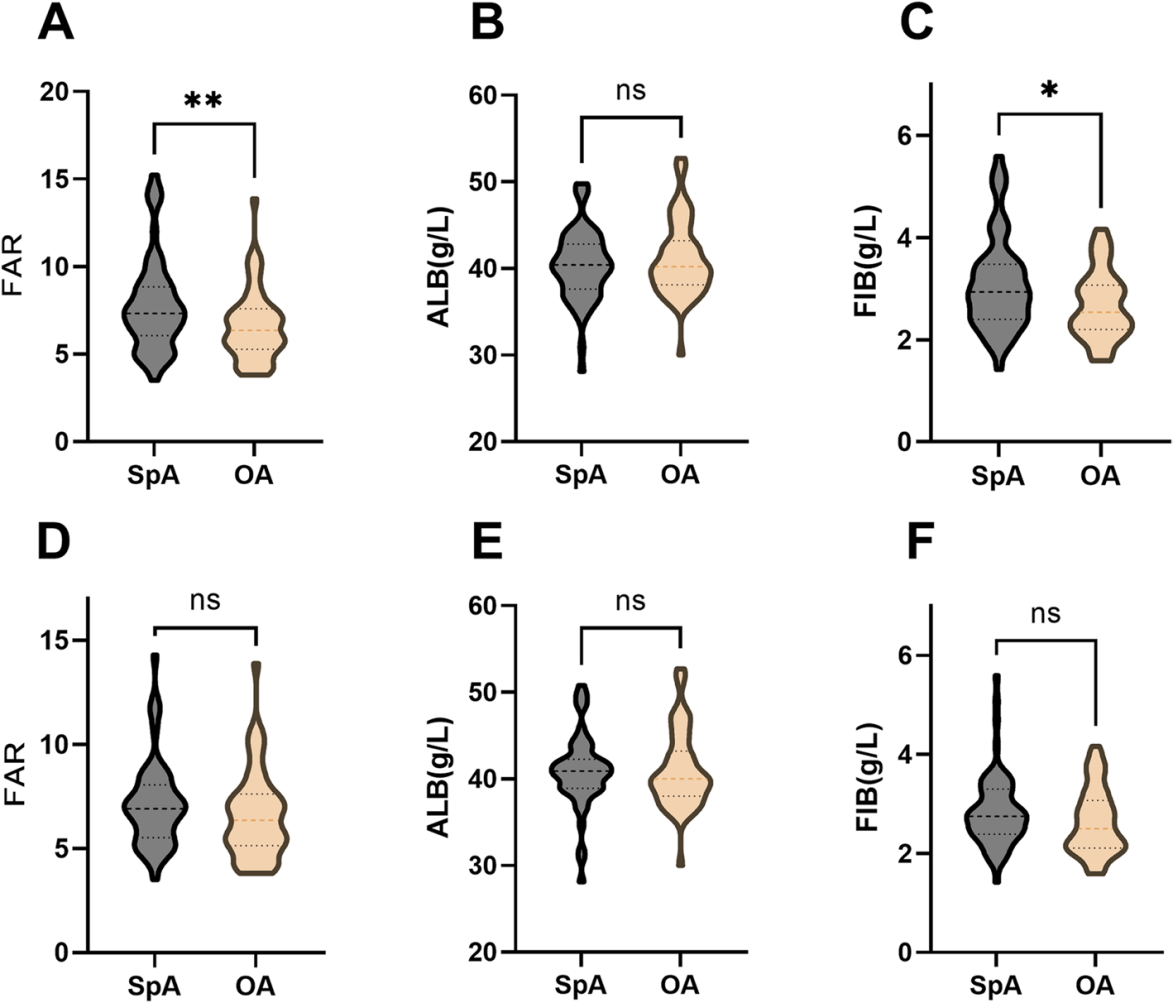
Comparisons of indicators between group spondyloarthritis and osteoarthritis after erythrocyte sedimentation rate and C-reactive protein matching. **A**-**C**, comparisons of fibrinogen to albumin ratio (**A**), albumin (**B**), and fibrinogen (**C**) after erythrocyte sedimentation rate matching. **D**-**F**, comparisons of fibrinogen to albumin ratio (**D**), albumin (**E**), and fibrinogen (**F**) after C-reactive protein matching. After ESR matching, FAR was still significantly higher in spondyloarthritis group than in osteoarthritis group (**A**). ALB: albumin; FIB: fibrinogen; FAR: fibrinogen to albumin ratio. *: *P*-value < 0.05; **: *P*-value < 0.01

For deeper analysis, ESR and CRP matching were imported to investigate whether there was a distinction between SpA group and OA group as well as within SpA subgroups when ESR or CRP was at the same level. Results came out that after ESR matching, FAR was still significantly higher in SpA group than OA group (*p* = 0.0083). After CRP matching, FAR level was also higher in the SpA group but not significant (*p* = 0.1266). FAR showed no significant difference within SpA subgroups after ESR and CRP matching. More information is exhibited in Fig. 1.

### Correlation between CRP, ESR and FIB, ALB, FAR

**Fig. 2:**
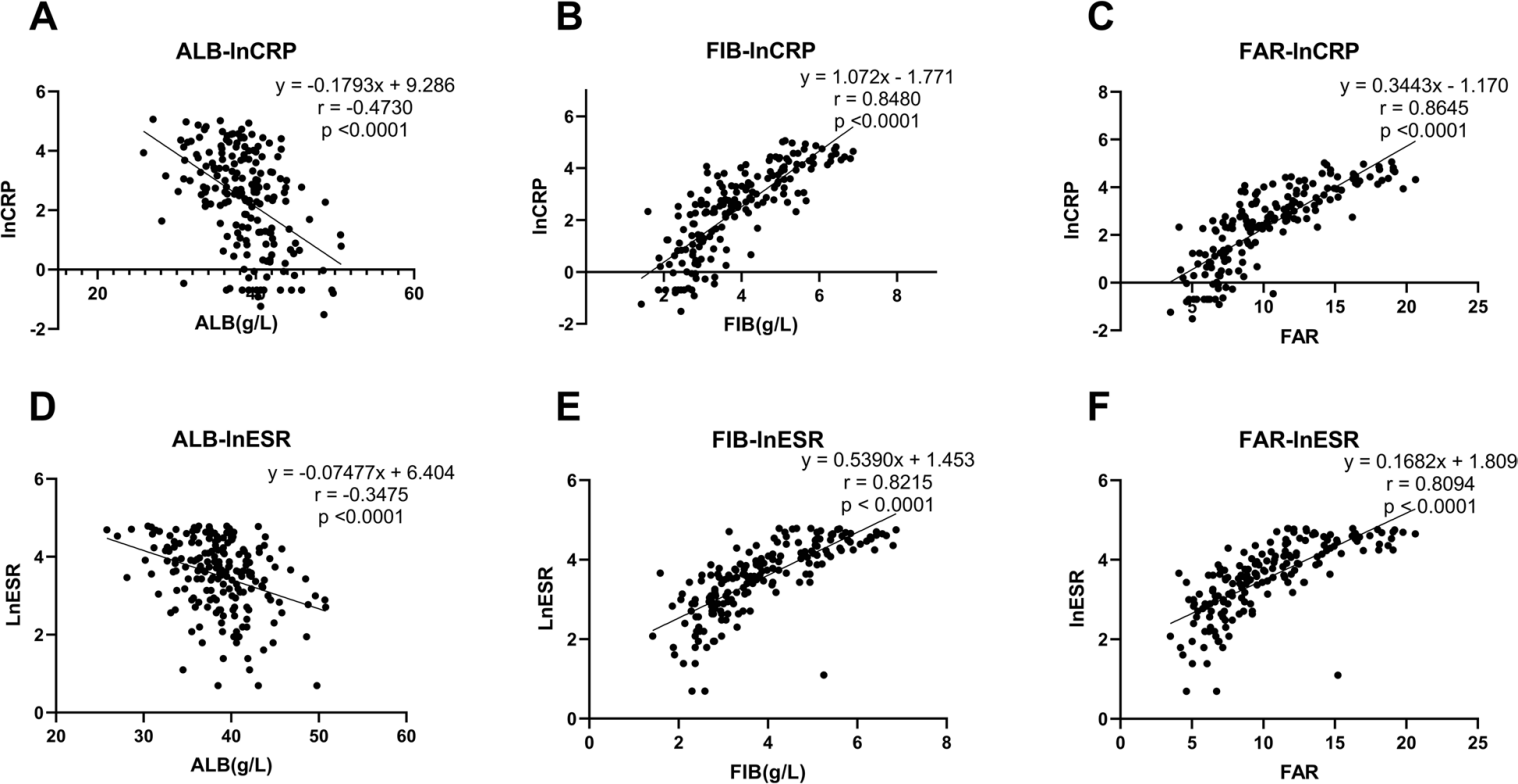
Correlation between conventional inflammatory markers and potential indicators. **A**-**C**, correlation between C-reactive protein and albumin (**A**), fibrinogen (**B**), fibrinogen to albumin ratio (**C**). D-F, correlation between erythrocyte sedimentation rate and albumin (**D**), fibrinogen (**E**), fibrinogen to albumin ratio (**F**). ALB: albumin; FIB: fibrinogen; FAR: fibrinogen to albumin ratio; Ln (CRP): the natural logarithm of C-reactive protein; Ln (ESR): the natural logarithm of erythrocyte sedimentation rate

Figure 2 exhibits the correlation of CRP-ALB, CRP-FIB, CRP-FAR, and ESR-ALB, ESR-FIB, ESR-FAR. Ln (CRP) and ln (ESR) were adopted in the calculation for preferable presentation. Analyses demonstrated that new indicators (ALB, FIB, and FAR) were significantly correlated to widely acknowledged markers (CRP and ESR) with all *P*-value < 0.0001. FIB and FAR showed positive correlation, while ALB was negatively correlated with CRP and ESR.

### Univariate and multivariate regression analyses

The regression analyses were applied for both CRP and ESR, as neither had an absolute advantage in clinical practice. WBC, ALB, FIB and FAR were significant factors for inflammation because the *P*-values of these four factors were all less than 0.0001 in univariate regression analysis. Then multivariate regression results showed that ALB, FIB, and FAR were all significant predictors for CRP, and only FIB and FAR were valuable indicators for ESR. WBC was excluded after multi-factor regression analyses, as the *P*-value of WBC was more than 0.05. However, the results of stepwise regression indicated that only FIB and FAR were significant predictors for CRP and ESR. Table 3 reported the slopes and their confidence intervals of the stepwise regression. A detailed process of regression analysis can be found in the supplementary document.

**Table 3:** Stepwise regression analysis

Regression formular	Slope	95% Confidence intervals	*P*-value
ESR ~ WBC + FAR			
WBC	1.63	0.19–3.07	0.025
FAR	6.41	5.59–7.23	< 2e-16
ESR ~ WBC + FIB + ALB			
WBC	1.52	0.08–2.96	0.036
ALB	−1.15	− 1.88- -0.42	0.0018
FIB	18.31	15.66–20.95	< 2e-16
CRP ~ FAR			
FAR	6.50	5.69–7.32	< 2e-16
CRP ~ ALB + FIB			
ALB	−1.24	−2.04- -0.45	0.0020
FIB	17.93	15.17–20.69	< 2e-16

(*WBC* white blood cells, *CRP* C-reactive protein, *ESR* erythrocyte sedimentation rate, *ALB* albumin, *FIB* fibrinogen, *FAR* fibrinogen to albumin ratio)

### Receiver operating characteristics analysis

**Fig. 3:**
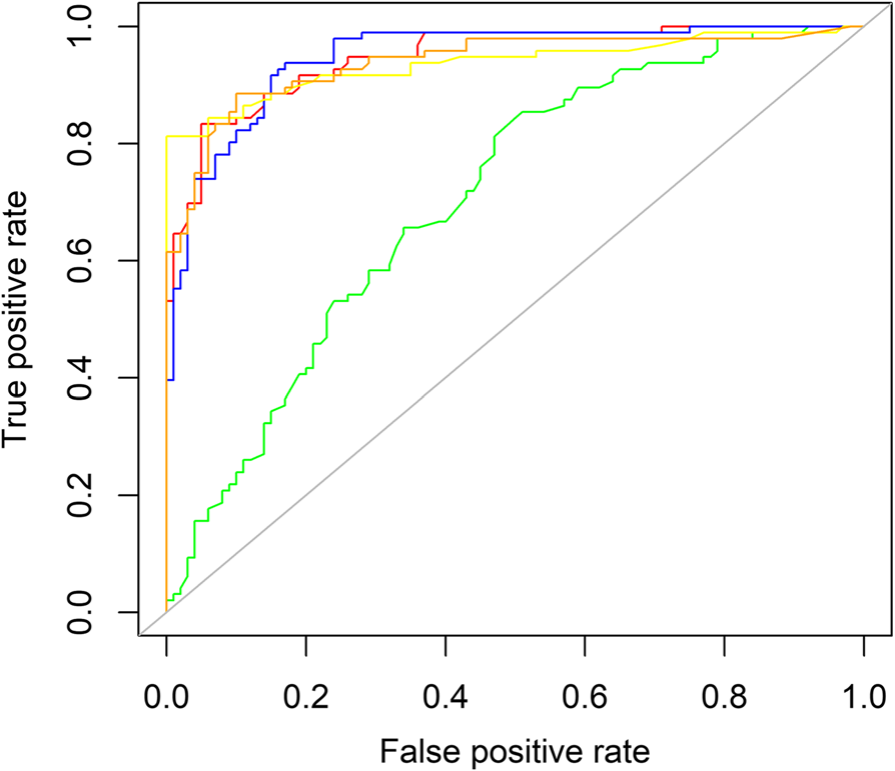
ROC curves of albumin, fibrinogen, fibrinogen to albumin ratio, C-reactive protein and erythrocyte sedimentation rate in identifying high inflammation state. Green: ROC curve of albumin; Red: ROC curve of fibrinogen; Blue: ROC curve of fibrinogen to albumin ratio; Yellow: ROC curve of erythrocyte sedimentation rate; Orange: ROC curve of C-reactive protein ROC: receiver operating characteristics

ALB, FIB and FAR were considered potential predictors of inflammation after multivariate regression analyses. After that, receiver operating characteristics analysis was reported to estimate the ability in identifying hyper-inflammatory state and to determine an appropriate cut-off value for the convenience of clinical judgment. Both FIB and FAR performed well in reflecting serious inflammation with the area under curve (AUC) of 0.95, but the AUC of ALB was just 0.67. A recommended cut-off value was 9.44 for FAR and 3.98 g/L for FIB, with the sensitivity of 0.91(FAR)&0.85(FIB) and specificity of 0.88(FAR)&0.94(FIB). Besides, a similar ROC analysis for ESR and CRP was also applied to show the ability of ESR and CRP compared to FIB and FAR. The AUC of ESR and CRP was 0.94, which was slightly less than FIB and FAR. A graphical representation of ROC analysis was displayed in Fig. 3. Additional ROC analysis was provided to explore the capacity of FAR and FIB to detect a slight inflammation. AUC of FAR was 0.90 and AUC of FIB was 0.88, which was lower than that in high inflammatory reaction, but still valuable. The suggested cut-off value was 8.34 for FAR and 3.38 g/L for FIB, while the specificity and sensitivity were 0.88&0.82 in FAR and 0.9&0.77 in FIB. More details are provided in Table 4.

**Table 4:** ROC analyses

Predictors	AUC_1_	Cut-off value_1_	LR + _1_	LR-_1_	AUC_2_	Cut-off value_2_	LR + _2_	LR-_2_
ALB	0.67	/	/	/	0.71	/	/	/
FIB	0.95	3.98	14.17	0.16	0.88	3.38	7.7	0.26
FAR	0.95	9.44	7.58	0.13	0.9	8.34	6.83	0.20
CRP	0.94	14.65	7.37	0.13	0.92	5.11	8.46	0.17
ESR	0.94	48	10.55	0.17	0.93	18	9.2	0.08

(1: ROC analysis in identifying high inflammation state; 2: ROC analysis in identifying slight inflammation state; *AUC* area under the curve, *LR+* positive likelihood ratio, *LR-* negative likelihood ratio, *ALB* albumin, *IB* fibrinogen, *FAR* fibrinogen to albumin ratio)

## Discussion

The first part of the study came out that FAR increased in the SpA group but remained lower in the OA and HC groups. In addition, the ReA group owned the highest FAR level among SpA subgroups (*p* < 0.05). Considering the consistency of CRP, ESR and FAR, the distinction can be explained by the fact that inflammation levels in spondyloarthritis patients are higher than in osteoarthritis patients and healthy controls. In previous studies, FAR or AFR (albumin to fibrinogen ratio) was proved to be related to a systemic inflammatory response in various malignant tumors, including but not limited to gastric, esophageal, lung and cervical cancers [17–20]. Another critical application of FAR was in cardiovascular diseases, while some studies revealed its predictive ability in hypertension, acute coronary syndrome, and ascending aortic aneurysm [21–23]. Furthermore, FAR was related to the severity and outcomes in some cardiovascular studies. A research showed the level of FAR was independently associated with the severity of coronary artery disease (CAD) [24]. Higher level of FAR forecasted worse outcomes among patients with CAD undergoing percutaneous coronary intervention (PCI) [25, 26]. Thus FAR is considered a general biomarker more than a specific predictor.

We then explored the relationship between FAR and conventional inflammatory biomarkers: CRP and ESR. FAR was significantly correlated to CRP and ESR. However, after ESR matching, FAR level in the SpA group is still significantly higher than in the OA group. The result may imply that the distinguishability of FAR was better than ESR, but the conclusion requires further evidence.

In order to discuss the potential role of FAR, the relationship between ALB, FIB and inflammation is vital. In the study, FIB was tied closer to CRP&ESR than ALB, but ALB also plays an important role in an inflammatory response. During an inflammatory response, synthesis rates of albumin elevate, but serum level of albumin decreases due to the increased microvascular permeability [27]. FIB, an essential part of the coagulation and fibrinolytic system, is not only shifted by inflammation, but also regulates inflammatory response. FIB is reported to be a ligand of several cell surface receptors, including VE-cadherin, ICAM-1, αIIbβ3, α5β1, αVβ3, αMβ2, and αXβ2, which enables FIB to affect leukocyte migration [28]. Furthermore, FIB can modulate leukocyte function by activating NF-kappa B transcription factors and stimulating macrophage chemokine secretion [29, 30]. It is worth noting that the ability of inflammatory regulation only occurs when fibrinogen is fixed on a surface or is converted into a polymer [28]. The fact suggests that a high level of plasma fibrinogen may be uninjurious in normal parts, but can affect the inflamed joints.

In addition to regulating inflammation, fibrinogen also participates in bone protection by binding to β2 integrin CD11b to restrain osteoclast precursors [31]. However, the protective effect is reversed by citrullination, and citrullinated fibrinogen may lead to osteoclasia [32]. Thus FAR may be considered a composite marker in connection with capillaries and coagulation function, reflecting inflammation from different sides of ESR and CRP.

The study also indicates that compared with ALB or FIB, FAR is considered more capable of assessing the severity of inflammation. Though the AUC of FIB is similar to FAR in ROC analysis, the difference of FAR is more significant than the difference of FIB between group SpA and OA. An interesting point on the curves signifies that the performance of FAR and FIB is different according to sensitivity intervals. Indeed, when the sensitivity is more than 0.9, the positive likelihood ratio (LR+) of FAR is higher than FIB under the same sensitivity. In contrast, FIB performs better when sensitivity is less than 0.9. For example, at a sensitivity of 0.91, the LR+ of FAR is 7.58 and the LR+ of FIB is 5.95. However, at a sensitivity of 0.85, the LR+ of FAR is 8.39 and the LR+ of FIB is 14.17. This phenomenon suggests that FAR was a better choice when high sensitivity is required.

The necessity of introducing a new inflammatory biomarker lies in the complicated situation in clinical practice: some patients present low ESR levels but show high CRP levels, or vice versa. In the study, 59 out of 196 patients with discordant for CRP and ESR were observed (details are provided in the supplementary document). The fact that the AUC of CRP and ESR was only 0.94 meant an inconsistency between CRP and ESR, which supported the situation. In comparison of CRP and ESR, FAR showed a similar ability to identify serious inflammation, which denoted that the ability of FAR is not inferior to the now widely used inflammatory indicators. Moreover, the application of FAR in assessing inflammation in spondyloarthritis can be cost-effective, as the detecting techniques of ALB and FIB are well developed and widely accepted in clinical practice. Compared to a brand new biomarker, FAR can be brought into use promptly.

In fact, the application of FAR in autoimmune diseases has been investigated in many previous studies. FAR was notably elevated in rheumatoid arthritis patients than in systemic lupus erythematosus patients, which could be proof that FAR is related to arthritis [33]. A study displayed the predictive ability of FAR in a cross-sectional study but failed to forecast a poor outcome in antineutrophil cytoplasmic antibody-associated vasculitis, which indicated that FAR is a marker of acute reaction [34]. Another study performed that FAR could help to distinguish the disease activity in AS [10].

This study expanded the target population to spondyloarthritis and osteoarthritis patients, which was not mentioned before. The correlation between FAR and traditional inflammatory biomarkers (CRP and ESR) as well as the capacity of FAR in identifying inflammation status supported FAR to be a new indicator in the assessment of inflammation in spondyloarthritis. Moreover, the difference of FAR between the SpA group and OA group after ESR matching suggested the potential of FAR. However, more profound research on the accuracy of CRP, ESR, and FAR in reflecting the disease activity of spondyloarthritis is required.

The study is limited to a retrospective and single-center design. Though multiple and regression interpolation has been employed to fill in missing data and healthy controls have been matched to SpA patients in age, gender, and WBC, there may still be an unestimated bias. Another limitation is that the study fails to assess the disease activity directly due to the disparate assessment criteria. In addition, the reliability of FAR may be questioned when a patient develops nephropathy, hepatopathy or hemopathy. Furthermore, according to the current study, FAR did not show more advanced abilities than ESR or CRP in reflecting inflammatory status. Whether FAR can perform better in other areas needs further research.

In summary, the study shows the ability of FAR in evaluating inflammation, which is related to ESR&CRP, but not limited to them. Ultimately, FAR is regarded as the most potential indicator, with the recommended cut-off value of 9.44 for serious inflammation and 8.34 for mild conditions compared to ALB and FIB. Further exploration of the direct relationship between FAR and SpA disease activity is expected, and an assessment standard including FAR is worth studying.

## Supplementary Information

**Additional file 1.**

## Data Availability

The datasets used or analyzed during the study are available from the corresponding author upon reasonable request.

## Abbreviations

FAR: Fibrinogen to albumin ratio
SpA: Spondyloarthritis
OA: Osteoarthritis
HC: Healthy controls
PsA: Psoriatic arthritis
IBDA: Arthritis related to inflammatory bowel disease
ReA: Reactive arthritis
JIA: Juvenile idiopathic arthritis
AS: Ankylosing spondylitis
NSAIDs: Non-steroidal anti-inflammatory drugs
ASDAS: Ankylosing Spondylitis Disease Activity Score
BASDAI: Bath Ankylosing Spondylitis Disease Activity Index
DAPSA: Disease Activity index for Psoriatic Arthritis
VAS: Visual Analogue Scale
CRP: C-Reactive protein
ESR: Erythrocyte sedimentation rate
ALB: Albumin
FIB: Fibrinogen
WBC: White blood cell
ROC: Receiver operating characteristics
AUC: Area under curve
AFR: Albumin to fibrinogen ratio
Ln (CRP): The natural logarithm of C-reactive protein
Ln (ESR): The natural logarithm of erythrocyte sedimentation rate
CAD: Coronary artery disease
PCI: Percutaneous coronary intervention
LR+: Positive likelihood ratio
LR-: Negative likelihood ratio
