# Supplementary material for: The potential value of fibrinogen to albumin ratio (FAR) in the assessment of inflammation in spondyloarthritis
Source: BMC Musculoskelet Disord. 2022 Sep 15;23:864. doi: 10.1186/s12891-022-05797-6 (PMC9479360; doi:10.1186/s12891-022-05797-6)
Supplement: Supplementary file 1 — Additional file 1. [file 12891_2022_5797_MOESM1_ESM.docx]

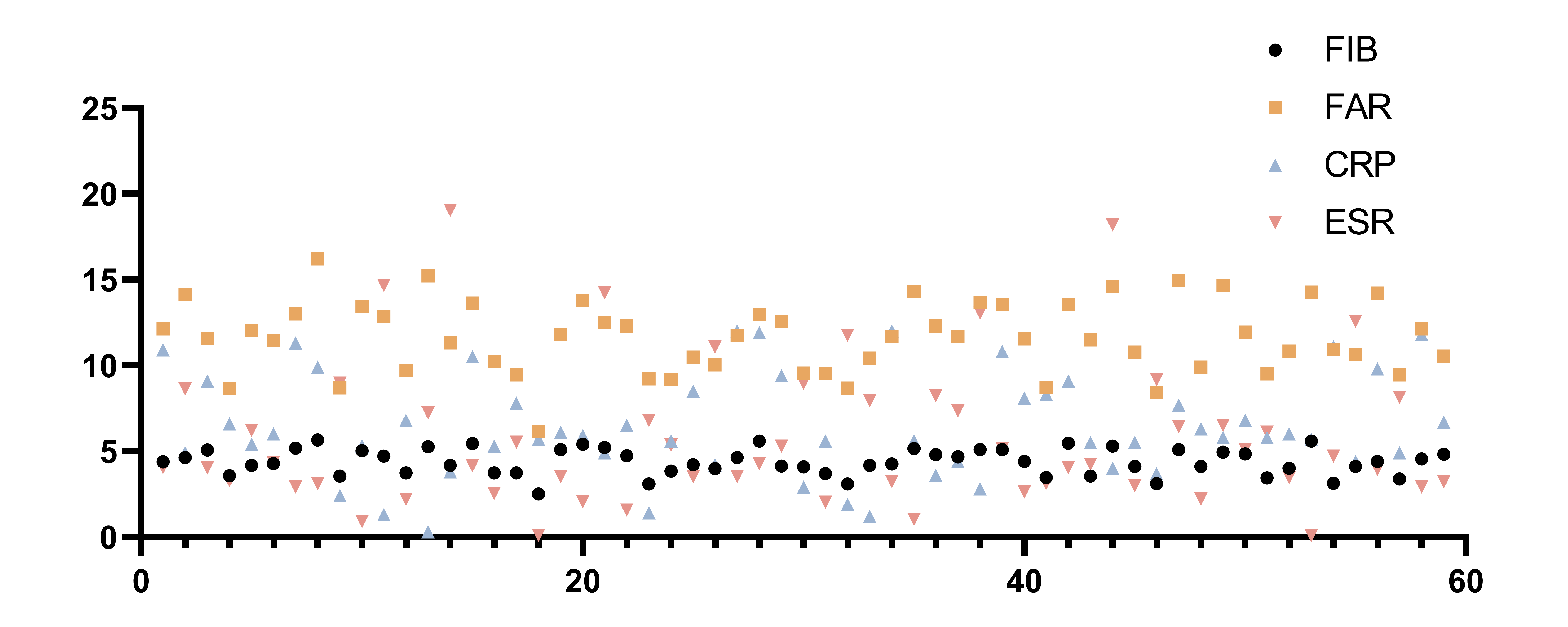


Supplementary Figure 1 FIB, FAR, CRP, ESR of the patients with discordant for CRP and ESR

(The value shown in the figure is 1/10 of the original ESR and 1/5 of the original CRP)

Univariate regression analysis

ESR

Estimate Std. Error Pr(>|t|)

age 0.1934 0.1454 0.185

gender -6.003 4.896 0.222

WBC 5.540 1.015 1.48e-07 *

ALB -3.1016 0.5053 4.62e-09 *

LYM -4.713 3.722 0.207

FIB 20.603 1.227 < 2e-16 *

FAR 6.7293 0.3877 < 2e-16 *

HLA -1.918 5.122 0.709

CRP

Estimate Std. Error Pr(>|t|)

age 0.2828 0.1448 0.0522

gender 0.8938 4.9196 0.856

WBC 4.247 1.048 7.35e-05 *

LYM -8.053 3.602 0.0267

ALB -3.0062 0.5089 1.53e-08 *

FIB 19.410 1.326 < 2e-16 *

FAR 6.5023 0.4081 < 2e-16 *

HLA -14.983 5.109 0.00382

Multivariate regression and stepwise regression analysis

1. ESR ~ WBC + FAR

Estimate Std. Error Pr(>|t|)

WBC 1.6302 0.7201 0.0247

FAR 6.4084 0.4090 < 2e-16 *

After Stepwise

ESR ~ WBC + FAR

AIC=1192.01

Estimate Std. Error Pr(>|t|)

WBC 1.6302 0.7201 0.0247

FAR 6.4084 0.4090 < 2e-16 *

1. ESR ~ WBC + FIB + ALB

Estimate Std. Error Pr(>|t|)

WBC 1.5217 0.7194 0.03570

ALB -1.1527 0.3639 0.00179

FIB 18.3061 1.3237 < 2e-16 *

After Stepwise

ESR ~ WBC + FIB + ALB

AIC=1191.5

Estimate Std. Error Pr(>|t|)

WBC 1.5217 0.7194 0.03570

ALB -1.1527 0.3639 0.00179

FIB 18.3061 1.3237 < 2e-16 *

1. CRP ~ WBC + FAR

Estimate Std. Error Pr(>|t|)

WBC 0.4287 0.7682 0.577

FAR 6.4953 0.4357 < 2e-16 *

After Stepwise

CRP ~ FAR

AIC=1215.26

Estimate Std. Error Pr(>|t|)

FAR 6.5023 0.4081 < 2e-16 *

1. CRP ~ WBC + FIB + ALB

Estimate Std. Error Pr(>|t|)

WBC 0.4086 0.7900 0.60564

FIB 17.9089 1.4548 < 2e-16 *

ALB -1.2393 0.4035 0.00244

After Stepwise

AIC=1225.72

CRP ~ ALB + FIB

Estimate Std. Error Pr(>|t|)

ALB -1.2435 0.3967 0.00199

FIB 17.9285 1.3806 < 2e-16 *

Regression analysis of FAR and FIB

Univariate regression analysis

FAR

Estimate Std. Error Pr(>|t|)

WBC 0.5971 0.1200 1.46e-06 *

HLA -1.7156 0.5696 0.00298

lym -0.8487 0.4234 0.0466

age 0.05068 0.01653 0.00248

gender -0.3445 0.5691 0.546

CRP 0.087171 0.005471 <2e-16 *

ESR 0.090393 0.005208 <2e-16 *

FIB

Estimate Std. Error Pr(>|t|)

WBC 0.19262 0.03872 1.45e-06 *

HLA -0.3653 0.1869 0.0522 .

lym -0.2034 0.1364 0.138

age 0.005433 0.005442 0.319

gender 0.06511 0.18355 0.723

CRP 0.027031 0.001847 <2e-16 *

ESR 0.028750 0.001712 <2e-16 *

ALB -0.09832 0.01938 9.14e-07 *

Stepwise regression of FAR and FIB

formula = FAR ~ ESR + CRP

AIC=247.02

Estimate Std. Error Pr(>|t|)

ESR 0.061551 0.004689 <2e-16 *

CRP 0.054897 0.004684 <2e-16 *

FIB

formula = FIB ~ ESR + CRP + ALB

Step: AIC=-167.41

Estimate Std. Error Pr(>|t|)

ESR 0.020688 0.001680 < 2e-16 *

CRP 0.017051 0.001668 < 2e-16 *

ALB 0.017107 0.011943 0.153676
